# Direct assessment of the antioxidant property of salivary histatin

**DOI:** 10.3164/jcbn.19-53

**Published:** 2019-10-08

**Authors:** Tomoko Komatsu, Kyo Kobayashi, Eva Helmerhorst, Frank Oppenheim, Masaichi Chang-il Lee

**Affiliations:** 1 Division of Dentistry for the Special Patient, Department of Critical Care Medicine and Dentistry, Kanagawa Dental University Graduate School of Dental Medicine, 82 Inaoka-cho, Yokosuka, Kanagawa 238-8580, Japan; 2 Yokosuka-Shonan Disaster Oral Health Research Center & Oxidative Stress/ESR Laboratories, Kanagawa Dental University Graduate School of Dental Medicine, 82 Inaoka-cho, Yokosuka, Kanagawa 238-8580, Japan; 3 Department of Molecular and Cell Biology, Boston University Henry M. Goldman School of Dental Medicine, Albany street, Boston, MA 02118, USA

**Keywords:** saliva, histatin, oxidative stress, reactive oxygen species, antioxidant

## Abstract

Histatin, a salivary protein, affects oral homeostasis through preservation of tooth integrity and protection against caries and fungal infections. However, the effects of histatin in the generation of oxidative stress induced by reactive oxygen species and in the oral cavity remain unclear. In this study, the effects of histatin on direct reactive oxygen species scavenging activity were examined using electron spin resonance. We demonstrated, for the first time, that histatin exhibits antioxidant activity against hydroxyl radicals generated by Fenton’s reaction by metal chelation or binding. The direct antioxidant effects of histatin, along with its antimicrobial activity, may be important in the oral protection of salivary proteins.

## Introduction

Salivary proteins play crucial roles in oral health as well as several lifestyle-related diseases through multiple host defense functions.^(1)^ These include homeostatic processes, lubrication, antimicrobial activity, and tooth demineralization/remineralization. These salivary elements may function as active indicators of both local and systemic disorders.^(2)^ Hyposalivation may also be a risk factor for acute respiratory infection.^(3)^ Because a number of salivary proteins have been identified, and their primary structures determined, it has become possible to explore their structure/function relationships. Saliva (oral fluid) is a biofluid with a perceived role in the protection of oral cavity surfaces against chemical, mechanical, and microbial attacks.^(4)^ Involved in this protective role is a complex mixture of proteins and peptides derived from salivary glands, gingival exudate, and cellular debris.^(5–9)^ Of these components, approximately 30% are low molecular weight proteins, commonly referred to as salivary peptides,^(10,11)^ which are assigned to four main classes: cystatins, histatins, statherin, and proline-rich proteins. These salivary peptides impact oral cavity homeostasis through preservation of tooth integrity,^(12)^ protection from dental caries,^(13)^ and avoidance of fungal infections.^(14)^

Reactive oxygen species (ROS) is a collective term for radical species of O_2_^•−^, HO^•^, nitric oxide, and non-radical oxygen derivatives. ROS production is a normal part of cellular metabolism. However, ROS overproduction disrupts tissue redox balance, inducing oxidative damage to DNA, lipids, and proteins.^(15,16)^ Increased ROS level or reduced antioxidant function, including ROS overproduction or impaired ROS removal, is referred to as oxidative stress, and may lead to several conditions.^(16–18)^ ROS is toxic to cells via enzyme inactivation, protein denaturation, DNA destruction, and lipid peroxidation.^(16–18)^ These events increase reactive aldehyde levels and lead to cell membrane damage.^(16–18)^

Oxidative stress is implicated in various lifestyle-related diseases, including atherosclerosis, myocardial infarction, cerebrovascular disease, diabetes mellitus, cancer, and osteoporosis.^(16–18)^ Furthermore, ROS causes loss of salivary antioxidant capacity, leading to the development of oral cancer in tobacco chewers and smokers.^(19,20)^ Antioxidant systems including antioxidant enzymes and antioxidants play a protective role by scavenging ROS.^(21–23)^

Histatin belongs to a family of slightly basic 3–4 kDa peptides containing multiple histidine residues.^(4,24)^ These peptides are secreted by parotid, submandibular, and sublingual glands, and were first characterized in the early 1970s as peptides that enhance the glycolytic activity of microorganisms.^(25,26)^ It was later reported that they have bactericidal and fungicidal properties.^(27,28)^ Structure-function studies on these proteins have identified distinct domains with specific functional properties. They display antifungal activity against a broad range of pathogens, including *Candida albicans*, *Cryptococcus neoformans*, and *Aspergillus fumigates*, and have antibacterial properties based on their killing and growth-inhibitory activity against various species of oral bacteria.^(29)^ The main human histatins are histatin 1, 3, and 5.^(30)^ Like other phosphorylated salivary proteins, histatin 1 is involved in the maintenance of tooth enamel mineral and pellicle formation.^(31)^ Among the histatins, histatin 5 displays the highest antifungal activity,^(30)^ and antifungal domains have been located in its N-terminal and middle regions. A segment spanning residues 4–15, designated P-113, has been evaluated for therapeutic efficacy in *in vivo* oral candidiasis.^(32,33)^ Recently, it has been reported that histatins 1 and 3, but not histatin 5, exhibit wound closure activities *in vitro*.^(34)^ The inactivity of histatin 5, comprising the 24 N-terminal residues of histatin 3, indicated that the C-terminal 8 residues in histatin 3 are essential for this activity. Because the last 7 of these 8 residues are homologous with the C terminus of histatin 1, this segment is possibly responsible for the wound-healing properties of histatins 1 and 3. Histatins also show affinity for mineral surfaces, reduce calcium phosphate precipitation, and maintain tooth integrity.^(35)^

The identification of functional regions within salivary proteins is critical to the development of artificial saliva. However, questions remain regarding the roles of salivary proteins, especially histatin, in ROS generation and oxidative stress in the oral cavity. Few studies have investigated the antioxidant effects of salivary proteins by measuring SOD level^(36,37)^ or lipid peroxidation.^(38)^ While the effect of copper-mediated oxidation of histatin 8 on the generation of HO^•^, as evaluated by electron spin resonance (ESR), has been reported,^(39)^ the direct effects of histatin on ROS generation have not been investigated. In the current study, we used ESR to investigate the effects of histatin on ROS scavenging effects. Our results provide the first direct evidence of the antioxidant properties of histatin.

## Materials and Methods

### Reagents

Xanthine oxidase (XO) [grade III: from buttermilk, chromatographically purified suspension in 2.3 M (NH_4_)_2_SO_4_, 10 mM sodium phosphate buffer (pH 7.8), containing 1 mM EDTA and 1 mM sodium salicylate], xanthine, and superoxide dismutase were obtained from Sigma (St. Louis, MO). Hydrogen peroxide (H_2_O_2_) and FeSO_4_ were obtained from Wako Chemical (Osaka, Japan). 5,5-Dimethyl-1-pyrroline-*N*-oxide (DMPO) was purchased from Labotec (Tokyo, Japan).

### Histatin

Synthetic histatin 1, 3, and 5 were obtained from the American Peptide Company (Sunnyvale, CA) and from Quality Controlled Biochemicals (Hopkinton, MA). Human parotid secretion (HPS) was collected from five healthy volunteers ranging in age from 25 to 38 years. Informed consent was obtained according to approved protocols of the Institutional Review Board at Boston University Medical Center. HPS was collected with a Curby cup device positioned over the orifice of the Stensen’s duct. HPS flow was stimulated with hard sour candies and the secretion was collected in graduated cylindrical tubes placed on ice.^(40)^ A 25-µl aliquot of PS was plated on blood agar (Hardy Diagnostics) to verify the sterility of the collected PS secretion (absence of water-soluble contamination). Dialyzed and lyophilized PS proteins were dissolved in buffer A consisting of 50 mM Tris/HCl and 50 mM NaCl, (pH = 8), applied to a MonoQ HR16/10 column (Amersham Biosciences, Uppsala, Sweden), and eluted at a flow rate of 2 ml/min with buffer B containing 50 mM Tris/HCl and 1 M NaCl (pH = 8) using the following gradient steps: 0–38 min: 0–13% buffer B; 38–233 min: 13–22% buffer B; 233–250 min: 22–40% buffer B. The purity of the synthetic histatins was verified by cationic, anionic PAGE, and reversed-phase analysis.^(41)^

### Determination of protein concentrations

Sample protein concentrations were measured using a micro-bicinchoninic acid (BCA) protein assay (Pierce Chemical, Co., Rockford, IL), with bovine serum albumin used as a protein standard.

### *In vitro* ESR measurement

HO^•^ was generated by the Fenton reaction (H_2_O_2_ plus FeSO_4_ or CuSO_4_) as described previously.^(21,22)^ The reaction mixtures comprised H_2_O_2_ (20 µM) and FeSO_4_ (20 µM) or CuSO_4_ (20 µM) in 0.1 M phosphate-buffered saline (pH 7.2) containing 50 mM 5,5-dimethyl-1-pyrroline-*N*-oxide (DMPO) as spin trap, with or without salivary protein pretreatment, respectively. Mixtures were transferred to a cell and the DMPO-OH spin adduct was measured using ESR.

For generation of HO^•^ by UV irradiation of H_2_O_2_, a reaction mixture containing 10 mM DMPO and H_2_O_2_ (20 mM) in 0.1 M phosphate-buffered saline (pH 7.2), with or without salivary protein pretreatment, was used. Mixtures were transferred to a cell and illuminated at 365 nm, 40 mW using a PAN UV lamp. After 20 s, the DMPO-OH spin adduct was measured with ESR.^(21,22)^

O_2_^•−^ was generated using the xanthine-XO system, as described previously.^(21,22)^ O_2_^•−^ was generated from XO (0.1 U/ml) and xanthine (362 µM) in 0.1 µM phosphate-buffered saline (pH 7.2) containing 50 mM DMPO with or without salivary protein pretreatment, respectively. The mixtures were transferred to a cell and the DMPO-OOH spin adduct was measured with ESR.

ESR observations were performed with a JES-RE 3X, X-band spectrometer (JEOL, Tokyo, Japan) connected to a WIN-RAD ESR Data Analyzer (Radical Research, Tokyo, Japan) at the following instrument settings: microwave power, 8.00 mW; magnetic field, 334.8 ± 5 mT; field modulation width, 0.079 mT; receiver gain, 400; sweep time, 1 min; and time constant, 0.03 s. Hyperfine coupling constants were calculated based on resonance frequency, measured with a microwave frequency counter, and on resonance field, measured with a JEOL ES-FC5 field measurement unit. ESR spectra were used to quantify the detected spin adducts for manganese oxide standards. After recording ESR spectra, their signal intensities, expressed as relative height, were normalized against the signal intensity of the manganese oxide standard.^(21,22)^ All experiments were repeated a minimum of four times.

### Statistical analysis

Statistical analyses were performed using Dunnett (OMS, Saitama, Japan). Data were tested for normality. Results are presented as mean ± SD. Two-way analysis of variance was used to compare the averages of three or four concentration levels. *P* values <0.05 were considered statistically significant.

## Results

### Effects of histatin 1, 3, and 5 on HO^•^ generation by the Fenton system

**Fig. 1:**
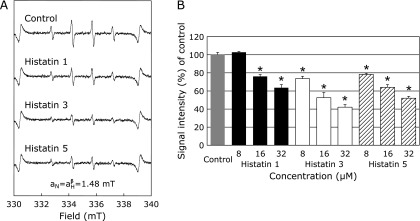
Effects of histatin 1, 3, and 5 (8, 16, and 32 µM) on HO^•^ generation from the Fenton reaction using FeSO_4_. (A) ESR spin trapping measurement of HO^•^ generated from H_2_O_2_ and FeSO_4_ in 0.1 M PBS, 50 mM DMPO as spin trap in the absence of histatin (control), and with histatin 1, 3, and 5 pretreatment at 32 µM, respectively. (B) Dose-response of histatins or control on HO^•^ generation from the Fenton reaction using FeSO_4_. *Significantly different (*p*<0.05) from the corresponding control value.

The effects of histatin 1, 3, and 5 on HO^•^ generated from the Fenton reaction were investigated by ESR spin trapping with DMPO. As reported previously,^(21,22)^ after adding H_2_O_2_ to FeSO_4_, a characteristic DMPO-OH spin adduct spectrum with hyperfine splitting giving rise to four resolved peaks was observed (Fig. 1A, control). Results indicate that the DMPO-OH signal was significantly reduced in a dose-dependent manner, except for the pretreatment of a final concentration of 8 µM of histatin 1 (Fig. 1A and B).

**Fig. 2:**
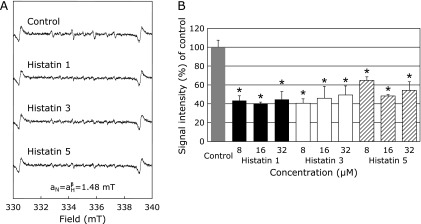
Effects of histatin 1, 3, and 5 (8, 16, and 32 µM) or control on HO^•^ generation from the Fenton reaction using CuSO_4_. (A) ESR spin trapping measurement of HO^•^ generation from H_2_O_2_ and CuSO_4_ in 0.1 M PBS, 50 mM DMPO as spin trap in the absence of histatin (control), or with histatin 1, 3, and 5 pretreatment at 32 µM, respectively. (B) Dose-response of histatins 1 or control on HO^•^ generation from the Fenton reaction using CuSO_4_. *Significantly different (*p*<0.05) from the corresponding control value.

The effects of histatin 1, 3, and 5 on HO^•^ generated using CuSO_4_, instead of FeSO_4_, were also investigated. Though the peaks were smaller than those generated using FeSO_4_, a characteristic DMPO-OH spin adduct spectrum with hyperfine splitting giving rise to four resolved peaks was observed following addition of H_2_O_2_ to CuSO_4_, (Fig. 2A, control). With histatin 1, 3, and 5 (8, 16, and 32 µM) pretreated with CuSO_4_ and subsequent addition of H_2_O_2_, the DMPO-OH signal was significantly reduced in comparison to the control (Fig. 2A and B).

### Effects of histatin 1, 3, and 5 on HO^•^ generation by ultraviolet irradiation of H_2_O_2_

**Fig. 3:**
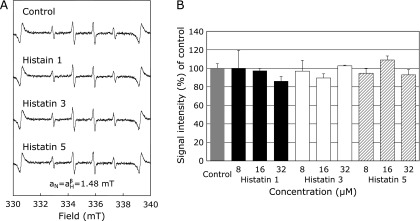
Effects of histatins 1, 3, and 5 (8, 16, and 32 µM) or control on HO^•^ generation from ultraviolet (UV) irradiation and H_2_O_2_. ESR spin trapping measurement of HO^•^ generation from UV irradiation and H_2_O_2_ in 0.1 M PBS, 50 mM DMPO as spin trap in the absence of histatins (control), or with histatin 1, 3, and 5 pretreatment at 32 µM, respectively. (B) The dose-response of histatins and control on HO^•^ generation from UV irradiation and H_2_O_2_ is represented.

The effects of histatin 1, 3, and 5 on HO^•^ generated from UV irradiation of H_2_O_2_ were investigated by ESR spin trapping with DMPO. As reported previously,^(21,22)^ following UV irradiation of H_2_O_2_, a characteristic DMPO-OH spin adduct spectrum with hyperfine splitting giving rise to four resolved peaks (Fig. 3A, control) was observed. When H_2_O_2_ was pretreated with histatin 1, 3, and 5, (8, 16, and 32 µM) and followed by UV irradiation, the DMPO-OH signal was not significantly reduced (Fig. 3A and B).

### Effects of histatin 1, 3, and 5 on O_2_^•−^ generation

**Fig. 4:**
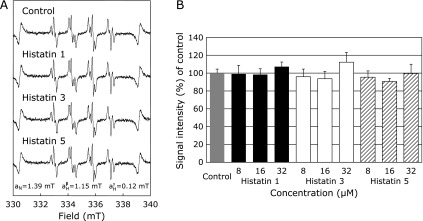
Effects of histatin 1, 3, and 5 (8, 16, and 32 µM) on O_2_^•−^ generation from xanthine oxidase (XO) and xanthine. (A) Electron spin resonance spin trapping measurement of O_2_^•−^ generation from XO (0.1 U/ml) and xanthine (362 µM) in 0.1 M PBS, 440 mM DMPO as spin trap in the absence of histatins (control), or with histatin 1, 3, and 5 pretreatment at a final concentration of 32 µM, respectively. (B) Dose-response of histatin 1, 3, and 5 or control on O_2_^•−^ generation from XO and xanthine.

 The effects of histatin 1, 3, and 5 on XO-mediated O_2_^•−^ generation were determined using ESR spin trapping with DMPO. As reported previously,^(21,22,42)^ after addition of xanthine to XO, a characteristic DMPO-OOH adduct spectrum with hyperfine splitting giving rise to 12 resolved peaks was observed (Fig. 4A). These signals were quenched by 150 U/ml superoxide dismutase, confirming that they were derived from O_2_^•−^ (data not shown). With histatin 1, 3, and 5 pretreatment of XO and subsequent addition of xanthine, however, alteration of the DMPO-OOH signal was not observed at any histatin concentration (Fig. 4A and B).

## Discussion

Histatin a human salivary protein, has antifungal activity and is susceptible to enzymatic degradation when released into the oral cavity^(43)^ Histatin 5 exhibits an antifungal effect by decreasing cell metabolism in *Candida albicans*.^(44,45)^ Histatin is unique to saliva and is rich in basic amino acid residues, which bind to the cell membranes of yeast and fungi, destroying their membrane structure. While mitochondrial-derived ROS is known to be involved in this antifungal action,^(43,44)^ the direct effect of histatin on ROS production has not been reported. In this study, we used ESR to evaluate the effect of histatin on ROS generation, showing that it reduced HO^•^ generation, but did not decrease O_2_^•−^ generation, from xanthine-XO (Fig. 1–4).

First, we confirmed that histatin 1, 3, and 5 suppressed HO^•^ generation by the Fenton reaction in a dose-dependent manner (Fig. 1A and C). Pathophysiological Fenton responses due to iron overload are reported by the ability of iron chelators, such as Desferal, to reduce high-intensity signals from DMPO-OH spin adducts, which are suggestive of HO^•^ formation.^(46,47)^ The production of HO^•^ from Fenton’s reaction in the living body is important in various ROS-induced diseases, including oral diseases.^(16,47,48)^

All histatins are enriched in histidine, an amino acid capable of complexing with divalent metal ions. Histatin is also activated by complexation of its three N-terminal amino acids (NH_2_-Asp-Ser-His-) with Cu^2+^ ions through modification of the ATCUN motif as metal binding site.^(49,50)^ Therefore, in order to investigate the effects on Cu^2+^ ion-mediated ROS production, we examined the effects of histatin on HO^•^ produced from Cu^2+^ and H_2_O_2_. Interestingly, histatin significantly inhibited HO^•^ generation (Fig. 2). Furthermore, in Fenton’s reaction with CuSO_4_, histatin 1, 3, and 5 suppressed the production of HO^•^, even at low concentrations (Fig. 2). These results suggest that histatin may have two physiological effects: antifungal action activated by Cu^2+^, and antioxidant effects by scavenging Cu^2+^ ion-related ROS.

In biological systems, homeostatic balance is maintained between ROS production and removal. This occurs even in the oral cavity, and disruption of this balance due to increased production of ROS increases the risk of oral disease.^(51,52)^ Additionally, oxidative stress due to balance modulation of ROS and antioxidants increased production of ROS related to oral diseases such as periodontitis, and to systemic diseases such diabetes and cardiovascular disease^(48,53)^ ROS is one of the most effective pathogenic mechanisms of chronic inflammation caused by bacteria, and undoubtedly leads to bone resorption.^(54,55)^ Neutrophils obtained from the peripheral blood of acute apical periodontitis (AAP) patients show increased production of ROS, particularly in response to treatment of chronic periapical granuloma.^(56)^ In addition, antioxidant salivary vitamins are known to be effective against oxidative stress caused by oral diseases such as oral lichen planus.^(57)^ We confirmed that histatin suppressed the production of HO^•^ from the Fenton reaction using FeSO_4_ or CuSO_4_ with H_2_O_2_ (Fig. 1 and 2). These results are the first direct evidence of the antioxidative effects by histatin. HO^•^ is generated by the biological Fenton reaction, as shown below in equations 1–3, and is critical in oxidative stress-induced diseases, including oral diseases.^(16,46,47)^ The finding that histatin scavenges HO^•^ produced by this reaction is an interesting evidence of a new role for the salivary oral defense system.

Fe^2+^ + H_2_O_2_ → Fe^3+^ + OH^−^ + HO^•^  (1)

Cu^2+^ + H_2_O_2_ → Cu^+^ + HOO^•^ + H^+^  (2)

Cu^+^ + H_2_O_2_ → Cu^2+^ + HO^−^ + HO^•^  (3)

We also investigated the effects of salivary proteins on other HO^•^ generating systems using UV irradiation of H_2_O_2_, a well-known Fe^2+^-independent reaction.^(21,22)^ Histatins did not affect HO^•^ generation by this system (Fig. 3). In our previous reports, Desferal, an iron chelator, reduced the high intensity signal of the ESR DMPO-OH spin adduct, which indicates the production of HO^•^.^(45,47)^ Therefore, combined with the results presented in Fig. 1–3, it appears that histatin’s HO^•^ scavenging effect is due to chelation of Fe^2+^ or Cu^2+^ ions, and not to direct scavenging of HO^•^ (Fig. 3), although the possibility of the oxidation of Fe^2+^ to Fe^3+^ by the reaction with histatin is not ruled out. In this study, it was confirmed that Fe^2+^ and Cu^2+^ inhibit HO^•^ formation via their chelating action (Fig. 2 and 3) by directly interacting with the Zn/Cu binding motif and/or amino acids within histatin 5.^(49,50)^ Further detailed studies on the influence of the formation of ROS by the reaction with histatin motif, amino acids, and metal ions are needed.

The amount of HO^•^ formed by CuSO_4_ was much smaller than that by FeSO_4_ (Fig. 1 and 2) because the rate constant of HO^•^ formation by Cu^+^ ions is larger than that by Cu^2+^ ions (equations 1–3). Although we should have used HO^•^ formation by Cu^+^ to examine the effect of histatin on HO^•^ formation in this study, we used the HO^•^ generation system using Cu^2+^ since it was reported that histatin has a binding site for divalent metal ions (Cu^2+^ and Zn^2+^).^(48,49)^ In the future, we would study the effects of histatin on HO^•^ formation, including the biokinetics of Cu (I, II).

Histatins did not affect O_2_^•−^ generation from XO and xanthine (Fig. 4). O_2_^•−^ and nitric oxide, either alone or through interaction, are responsible for the physiological, pathophysiological, biological role *in vivo*. Histatin does not directly scavenge O_2_^•−^, but reduces HO^•^ generation by Fenton’s reaction (Fig. 1, 2, and 4). While O_2_^•−^, which plays a critical physiological role and has weak oxidizing power, was not eliminated, histatin displayed excellent antioxidant effects against HO^•^ (Fig. 1–4). This showed that histatin functions as an effective defense against oral oxidative stress. Saliva is known to possess a variety of physiologically active substances, some of which have bactericidal effects. The antimicrobial action of histatin could be linked to the antioxidant effect observed in this study (Fig. 1–4).

In conclusion, ESR was used to demonstrate, for the first time, that histatin 1, 3, and 5 display antioxidant activity against HO^•^ by chelation or direct binding of Fe^2+^ or Cu^2+^. Direct evidence of histatin’s effects suggest that the antioxidant action of salivary protein may be important to antibacterial action in the oral cavity.
